# TRADD regulates perinatal development and adulthood survival in mice lacking RIPK1 and RIPK3

**DOI:** 10.1038/s41467-019-08584-5

**Published:** 2019-02-11

**Authors:** John P. Dowling, Mohamed Alsabbagh, Christina Del Casale, Zheng-Gang Liu, Jianke Zhang

**Affiliations:** 1 Department of Microbiology and Immunology, Sidney Kimmel Cancer Center, Thomas Jefferson University, 233 S. 10th St, Room 731, Philadelphia, PA 19107 USA; 2 Center for Cancer Research, National Cancer Institute, 37 Convent Drive, Bethesda, MD 20892 USA

## Abstract

TRADD is an adaptor for TNFR1-induced apoptosis and NFκB activation. However, TRADD-deficient mice undergo normal development and contain normal lymphoid populations, which contrasts with an embryonic defect in mice lacking FADD, the shared adaptor mediating apoptosis. Recent studies indicate FADD suppresses embryonic necroptosis mediated by RIPK1. TRADD was suggested to also mediate necroptosis. Here we report that targeting TRADD fails to rescue *Fadd*^*−/−*^ embryos from necroptosis, and ablation of TRADD rescues *Ripk1*^*−/−*^ mice from perinatal lethality when RIPK3-mediated necroptosis is disabled. The resulting *Ripk1*^*−/−*^*Ripk3*^*−/−*^*Tradd*^*−/−*^ mice survive until early adulthood, but die thereafter. A single allele of *Tradd* is optimal for survival of *Ripk1*^*−/−*^*Ripk3*^*−/−*^*Tradd*^*+/−*^ mice. We show that TRADD plays a more dominating role in NFκB-signaling than RIPK1. While RIPK1 protects thymocytes from TNFα-induced apoptosis, TRADD promotes this process. The data demonstrate that TRADD is critical in perinatal and adult mice lacking RIPK1 and RIPK3, which has not been appreciated in prior studies.

## Abstract (web summary)

Programmed cell death regulates early development but how various factors (for example, TRADD, FADD and RIPKs) regulate this is unclear. Here, the authors show that a single allele of *Tradd* is essential for survival, when both *Ripk3* and *Ripk1* are knocked out in mice, and RIPK1 protects thymocytes from TNF-induced apoptosis.

## Introduction

Programmed cell death (PCD) including apoptosis and necroptosis plays an important role during development^1,2^. In the immune system, PCD is required for homeostasis and suppression of autoimmunity^3,4^. Signaling through death receptors (DRs) of the TNFR1/Fas family can lead to PCD^5^. Apoptosis is mediated by the Fas associated death domain (FADD) adaptor protein, which recruits and activates caspase 8 to trigger the apoptotic program^6–11^. Apoptotic cells are engulfed by phagocytic cells, preventing spillage of intracellular contents, and thus limiting tissue damage and inflammation^12–14^. When apoptosis is blocked, necroptosis (or programmed necrosis) is initiated by receptor interacting protein kinase (RIPK)1 and RIPK3^15–19^. These two protein serine/threonine kinases interact with one another via their RIP homotypic interaction motif (RHIM). This results in phosphorylation of both RIPK1 and RIPK3 and recruitment/activation of the mixed lineage kinase domain like (MLKL) protein. Activated MLKL translocates to and disrupts the plasma membrane^20,21^. Loss of membrane integrity during necroptosis results in the release of cellular contents, leading to inflammatory responses^22^.

Although FADD and Caspase 8 were initially characterized as mediators of apoptotic cell death, genetic studies demonstrated these proteins paradoxically play a major role in preventing necroptotic cell death. *Fadd*^*−/−*^ embryos and *Casp8*^*−/−*^ embryos die in utero and do not survive past embryonic day (E)10.5^23–25^. Deletion of RIPK1 or RIPK3 corrects the embryonic defect in *Fadd*^*−/−*^ or *Casp8*^*−/−*^ mice^26–28^. Furthermore, deletion of RIPK3 completely restores normal development of *Fadd*^*−/−*^ or *Casp8*^*−/−*^ mice^27–29^. The resulting *Casp8*^*−/−*^*Ripk3*^*−/−*^ or *Fadd*^*−/−*^*Ripk3*^*−/−*^ double knockout (DKO) mice develop progressive lymphadenopathy and splenomegaly, a hallmark of lymphoproliferative (*lpr*) diseases.

Although RIPK1 (RIP or RIP1) was originally cloned as a Fas-interacting protein, deletion of RIPK1 results in perinatal lethality^30^, while Fas is dispensable in mouse development. Moreover, conditional deletion of RIPK1 specifically in T cells does not affect Fas-induced apoptosis^31^. *Ripk1*^*−/−*^ mice survive into late adulthood only when apoptosis is blocked by ablation of FADD or Caspase 8 and necroptosis is blocked by ablation of RIPK3^29,32–34^. When only necroptosis is blocked, *Ripk1*^*−/−*^*Ripk3*^*−/−*^ mice die within several days after birth. TRADD is the primary adaptor for TNFR1 and can induce both apoptosis and NFκB activation^35,36^. However, *Tradd*^*−/−*^ mice are viable and show no apparent developmental abnormality, despite defective TNFR1-mediated apoptosis and NFκB and MAPK signaling in *Tradd*^*−/−*^ cells^37–39^. TRADD also mediates apoptosis and activation of NFκB and MAPK signaling through toll-like receptor (TLR)3 and TLR4 as well as other DRs^38,40^. It has been suggested that TRADD, like RIPK1, can mediate necroptosis^38^. A better understanding of how TRADD fits into current models of cell death induction and regulation is essential to uncover a more complete picture of apoptotic and necroptotic signaling.

Here, we report an approach to investigate the physiological function of TRADD by employing unique animal models. The data indicates that TRADD plays no role in embryonic necroptosis in *Fadd*^*−/−*^ mice. However, the study uncovers a critical function for TRADD in mouse perinatal development as well as in adult mouse survival in the absence of RIPK1 and RIPK3. Moreover, TRADD is essential for lymphocyte survival in adult mice. The data show that TRADD mediates both apoptosis and NFκB activation. A single allele of TRADD is optimal for survival of of *Ripk1*^*−/−*^*Ripk3*^*−/−*^ mice, indicating a Goldilocks principle.

## Results

### TRADD is dispensable for necroptosis in mouse embryos

In agreement with previous analysis of *Casp8*^*−/−*^ mice^33^, we found that lack of TNFα improves the survival of *Fadd*^*−/−*^ embryos (Supplementary Fig. 1), indicating that TNFR1-mediated necroptosis is partially responsible for embryonic death. TRADD is suggested to be the primary adaptor for TNFR1 and likely mediates both apoptosis and NFκB activation^35,36^. However, *Tradd*^*−/−*^ mice exhibit no apparent defect in development and contain a normal hematopoietic compartment^37–39^. TRADD was also suggested to mediate TNFα-induced necroptosis^38^. However, we found that absence of TRADD failed to rescue *Fadd*^*−/−*^ embryos from death (Supplementary Fig. 2).

*Ripk1*^*−/−*^ or *Ripk1*^*−/−*^*Fadd*^*−/−*^ mice undergo normal embryogenesis but die within one day of birth due to RIPK3-mediated necroptosis^26,30^. In contrast, *Ripk1*^*−/−*^*Fadd*^*−/−*^*Ripk3*^*−/−*^ mice are viable, indicating that RIPK3-mediated necroptosis is the leading cause of perinatal lethality in *Ripk1*^*−/−*^*Fadd*^*−/−*^ mice^29,32–34^. Given possible functional redundancy between TRADD and FADD for apoptotic signaling and potential functional redundancy between TRADD and RIPK1 for NFκB signaling, we analyzed the impact of TRADD inactivation on the survival of *Ripk1*^*−/−*^ or *Ripk1*^*−/−*^*Fadd*^*−/−*^ mice. As shown in Supplementary Fig. 3, all *Ripk1*^*−/−*^*Tradd*^*−/−*^ mice died within one day of birth and were indistinguishable from *Ripk1*^*−/−*^ mice, indicating that deletion of TRADD has no survival benefit in *Ripk1*^*−/−*^ mice. In addition, intercross of *Ripk1*^*+/−*^*Fadd*^*+/−*^*Tradd*^*−/−*^ mice yielded no viable *Ripk1*^*−/−*^*Fadd*^*−/−*^*Tradd*^*−/−*^ mice at weaning age (Supplementary Fig. 3a, c), unlike the normal postnatal development seen in *Ripk1*^*−/−*^*Ripk3*^*−/−*^*Fadd*^*−/−*^ mice described in prior studies^29,32–34^. In total, the data indicate that TRADD does not mediate lethal necroptosis in *Fadd*^*−/−*^ embryos or in *Ripk1*^*−/−*^ and *Ripk1*^*−/−*^*Fadd*^*−/−*^ neonates.

### TRADD deletion in *Ripk1*^*−/−*^*Ripk3*^*−/−*^ mice

**Fig. 1:**
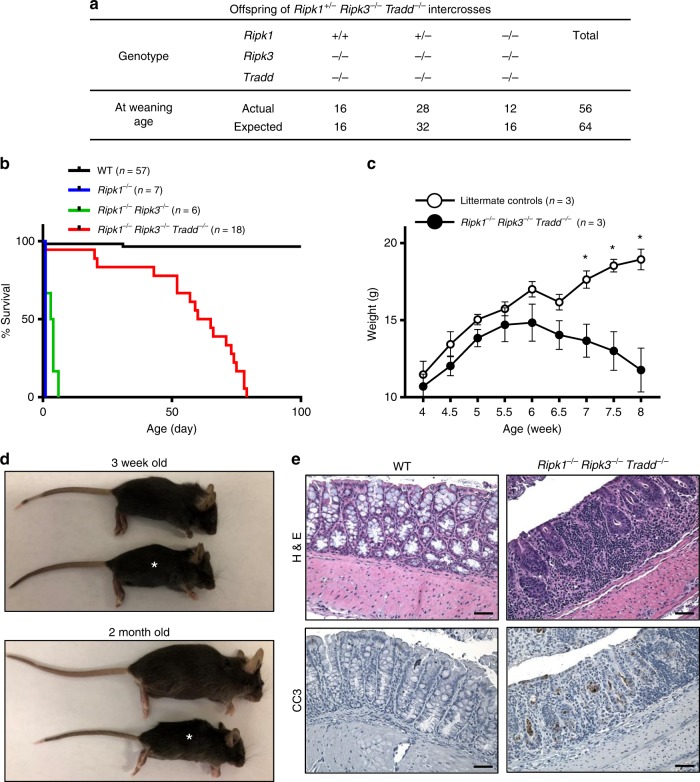
Deletion of TRADD improves the survival of *Ripk1*^*−/−*^*Ripk3*^*−/−*^ into young adulthood. **a** Actual and expected frequencies of pups at weaning age from intercrosses of *Ripk1*^*+/−*^*Ripk3*^*−/−*^ *Tradd*^*−/−*^ mice. Chi square analysis shows no significant difference between actual and expected (*p* = 0.472). **b** Kaplan–Meier plot showing survival of mice of indicated genotypes. Logrank tests shows a significant increase in survival of *Ripk1*^*−/−*^ *Ripk3*^*−/−*^ *Tradd*^*−/−*^ mice, compared to *Ripk1*^*−/−*^ *Ripk3*^*−/−*^ mice (*p* < 0.0001) but significantly less than WT mice (*p* < 0.0001). **c** Weights of *Ripk1*^*−/−*^ *Ripk3*^*−/−*^ *Tradd*^*−/−*^ mice and littermate controls over time presented as mean ± SEM, *p < 0.05 as determined by Student’s *t* test. **d** Three week old and two month old *Ripk1*^*−/−*^ *Ripk3*^*−/−*^ *Tradd*^*−/−*^ mice (*) with littermate controls (*n* = 5 independent experiments). **e** Hematoxylin and eosin (H&E) and cleaved caspase 3 (CC3) staining of the large intestine of indicated genotypes (*n* = 3 independent experiments). Scale bars are 50 μm

We performed further analysis to evaluate the in vivo function of TRADD in apoptosis using *Ripk1*^*−/−*^*Ripk3*^*−/−*^ mice, in which necroptosis is blocked. To this end, viable *Ripk1*^*+/−*^*Ripk3*^*−/−*^*Tradd*^*−/−*^ mice were generated and intercrossed. Weaning age *Ripk1*^*−/−*^*Ripk3*^*−/−*^*Tradd*^*−/−*^ triple knockout (TKO) mice were detected at expected Mendelian frequencies (Fig. 1a), which is in sharp contrast to the perinatal lethality exhibited by *Ripk1*^*−/−*^*Ripk3*^*−/−*^ neonates (Fig. 1b). This data provides in vivo evidence that a TRADD-mediated signaling pathway blocks perinatal mouse development in the absence of RIPK1 and RIPK3. However, *Ripk1*^*−/−*^*Ripk3*^*−/−*^*Tradd*^*−/−*^ mice fail to thrive in adulthood and progressively lose weight over a period of several weeks (Fig. 1b–d). Histological analysis of the *Ripk1*^*−/−*^*Ripk3*^*−/−*^*Tradd*^*−/−*^ large intestine showed an obvious loss of goblet cells and massive immune cell infiltration in the TKO intestine (Fig. 1e). In addition, elevated levels of activated caspase 3 (CC3) were evident in the large intestine of the *Ripk1*^*−/−*^*Ripk3*^*−/−*^*Tradd*^*−/−*^ TKO mice, when compared to wild-type control mice (Fig. 1e). Among the mice analyzed, the longest survival is postnatal day (P)78, when the moribund animal was euthanized (Fig. 1b).

**Fig. 2:**
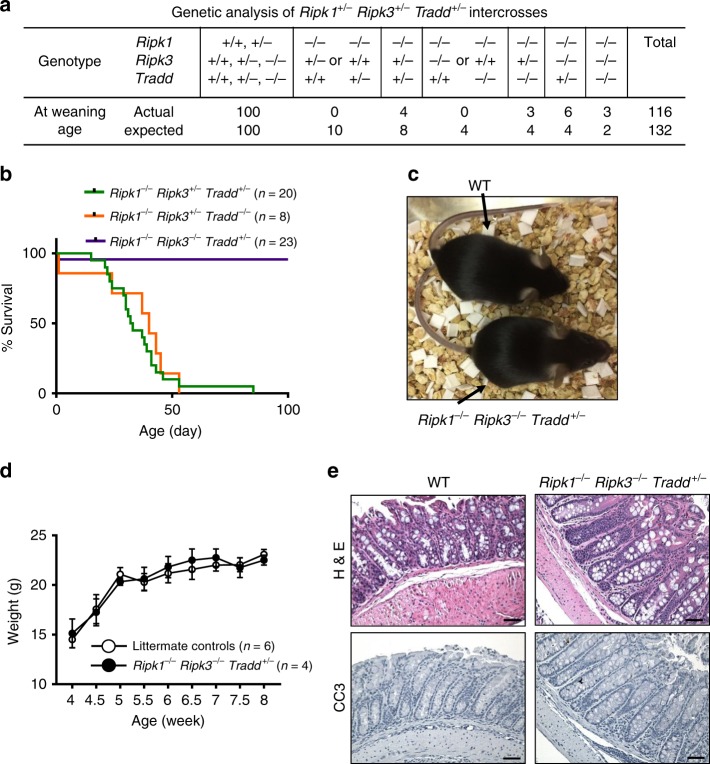
Haplosufficiency of TRADD rescues the lethality of *Ripk1*^*−/−*^*Ripk3*^*−/−*^ mice. **a** Expected and actual frequencies of pups at weaning age from a triple heterozygous cross of *Ripk1*^*+/−*^*Ripk3*^*+/−*^*Tradd*^*+/−*^ mice. **b** Kaplan-Meier plot showing survival of mice of indicated genotypes. Logrank tests showed *Ripk1*^*−/−*^*Ripk3*^*−/−*^*Tradd*^*+/−*^ mice exhibited improved survival over *Ripk1*^*+/−*^*Ripk3*^*−/−*^*Tradd*^*+/−*^ and *Ripk1*^*−/−*^*Ripk3*^*+/−*^*Tradd*^*−/−*^ mice (*p* < 0.0001). **c** One month old mice of indicated genotypes (*n* = 5 independent experiments). **d** Weights of *Ripk1*^*−/−*^*Ripk3*^*−/−*^*Tradd*^*+/−*^ mice and littermate controls over time presented as mean ± SEM. *t* test shows no significant difference between groups. **e** H&E and cleaved caspase 3 (CC3) staining of the large intestine of indicated genotypes (*n* = 3 independent experiments). Scale bars are 50 μm

To understand the role of TRADD in adult mice further, analysis was performed with intercross of triple heterozygous (THZ) *Ripk1*^*+/−*^*Ripk3*^*+/−*^*Tradd*^*+/−*^ mutant mice. Interestingly, heterozygosity of both RIPK3 and TRADD was sufficient for *Ripk1*^*−/−*^ mice to survive from birth to weaning age (Fig. 2a). Among the resulting *Ripk1*^*−/−*^*Ripk3*^*+/−*^*Tradd*^*+/−*^ mutant mice analyzed, survival up to P85 was observed, when the moribund animal was euthanized (Fig. 2b). Remarkably, retaining just one wild-type allele of the *Tradd* gene provides better survival than two wild-type alleles (*Tradd*^+/+^) or two KO alleles (*Tradd*^*−/−*^) for adult *Ripk1*^*−/−*^*Ripk3*^*−/−*^ mice (Fig. 2a–c). Weaning age *Ripk1*^*−/−*^*Ripk3*^*−/−*^*Tradd*^*+/−*^ mice are found at expected Mendelian frequencies (Fig. 2a) and survived normally throughout adulthood (Fig. 2b), in contrast to *Ripk1*^*−/−*^*Ripk3*^*−/−*^*Tradd*^*−/−*^ mice, which die in early adulthood (Fig. 1b). *Ripk1*^*−/−*^*Ripk3*^*−/−*^*Tradd*^*+/−*^ mice are indistinguishable in appearance and weight from wild-type controls (Fig. 2c, d), and are fertile. In addition, *Ripk1*^*−/−*^*Ripk3*^*−/−*^*Tradd*^*+/−*^ mice were similar to wild-type mice with minimal levels of activated caspase 3 in the large intestine (Fig. 2e), unlike *Ripk1*^*−/−*^*Ripk3*^*−/−*^*Tradd*^*−/−*^ mice, which show greatly elevated active caspase 3 levels in the large intestine (Fig. 1e). *Ripk1*^*−/−*^*Ripk3*^*−/−*^*Tradd*^*+/−*^ mice older than 6 months display no enlargement of the lymphoid organs (Supplementary Fig. 4a) nor presence of the abnormal CD3^+^B220^+^ population (Supplementary Fig. 4b), the typical symptoms of lymphoproliferative (*lpr*) disease present in *Fas*^*−/−*^ mice. This contrasts the severe *lpr* phenotype in *Fadd*^*−/−*^*Ripk3*^*−/−*^ or *Fadd*^*−/−*^*Ripk3*^*−/−*^*Ripk1*^*−/−*^ mice shown previously by others and us (Supplementary Fig. 4)^29,32–34^.

### A T cell defect in adult *Ripk1*^*−/−*^Ripk3^*−/−*^*Tradd*^*−/−*^ mice

**Fig. 3:**
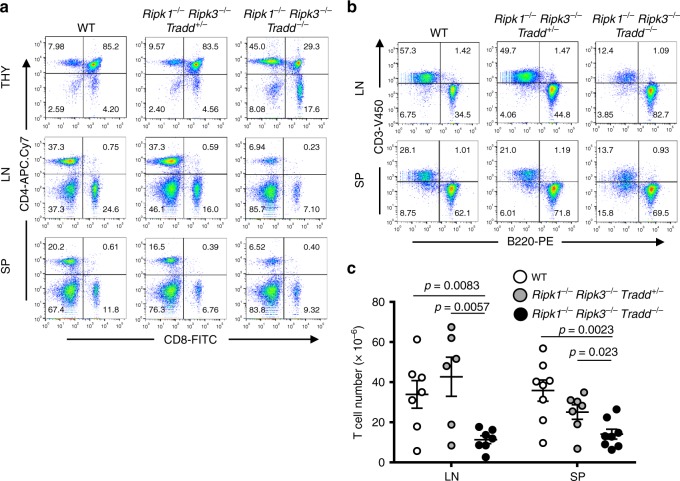
*Ripk1*^*−/−*^*Ripk3*^*−/−*^*Tradd*^*−/−*^ mice display impaired T cell development and T lymphopenia in young adulthood. **a**, **b** Representative two-color flow cytometric plots showing **a** CD4^+^ and CD8^+^ T cells in the lymph nodes, spleen, and thymus and **b** T cells (CD3^+^) and B cells (B220^+^) in the lymph nodes and spleen (*n* = 8 independent experiments). **c** CD3^+^ T cell numbers in the lymphoid organs of 7–8 week old mice of indicated genotypes (*n* = 7 independent experiments). All graphs presented as mean ± SEM

*Tradd*^*−/−*^ mice contain normal lymphocyte populations, indicating a dispensable role for TRADD in lymphopoiesis^38^. Here, we show that in young adult mice (7–8 week old), the lymphoid organs of *Ripk1*^*−/−*^*Ripk3*^*−/−*^*Tradd*^*+/−*^ mice are indistinguishable from wild-type controls despite a reduction of TRADD protein levels and the absence of RIPK1 and RIPK3 (Supplementary Fig. 5). However, deletion of both TRADD alleles results in an enlarged spleen in *Ripk1*^*−/−*^*Ripk3*^*−/−*^*Tradd*^*−/−*^ mice (Supplementary Fig. 5b, c). Flow cytometric analysis indicates that the spleen of *Ripk1*^*−/−*^*Ripk3*^*−/−*^*Tradd*^*−/−*^ mice contain an increased Mac^+^Gr1^+^ myeloid cell population (Supplementary Fig. 6,a). The thymus is significantly reduced by size and weight (Supplementary Fig. 5b, c) and thymic cellularity was also greatly decreased compared with WT controls (Supplementary Fig. 5d). Flow cytometric analysis showed a major reduction of the immature CD4^+^CD8^+^ double positive population in the thymus of *Ripk1*^*−/−*^*Ripk3*^*−/−*^*Tradd*^*−/−*^ mice (Fig. 3a), indicating impaired T cell development. Further analysis of the peripheral lymphoid organs showed that the spleen and lymph nodes exhibited a marked reduction of CD3^+^ T cell numbers of both CD4^+^ and CD8^+^ lineages (Fig. 3a–c), resulting in a dramatic T lymphopenia. In addition, *Ripk1*^*−/−*^*Ripk3*^*−/−*^*Tradd*^*−/−*^ mice have significantly reduced bone marrow cell numbers (Supplementary Fig. 5d). Flow cytometric analysis of the B lineage showed a decrease in B220^lo^IgM^−^ pro/pre B cells in the bone marrow and no apparent defect in the periphery in *Ripk1*^*−/−*^*Ripk3*^*−/−*^*Tradd*^*−/−*^ mice (Supplementary Fig. 6b, c).

### TRADD mediates apoptosis in T cells

**Fig. 4:**
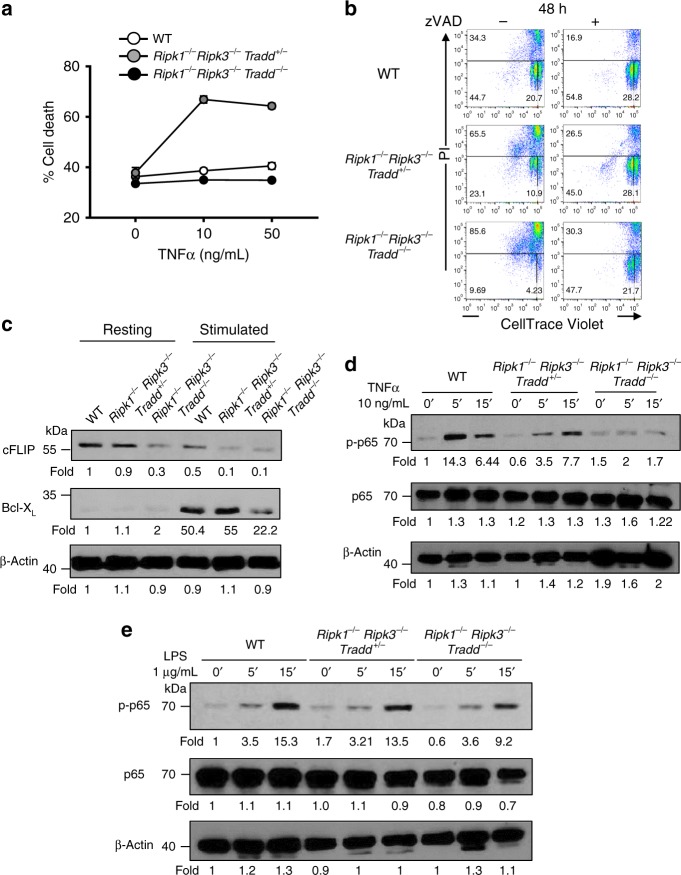
Cell death and survival of *Ripk1*^*−/−*^*Ripk3*^*−/−*^*Tradd*^*+/−*^ and *Ripk1*^*−/−*^*Ripk3*^*−/−*^*Tradd*^*−/−*^ cells. **a** Thymocytes were treated with indicated concentrations of TNFα for 16 h with 30 μg/mL cycloheximide. After addition of 1 μg/mL propidium iodide (PI), dead cells (PI^+^) were determined by flow cytometry (performed in triplicate, presented as mean ± SEM, *n* = 3 independent experiments). **b** Mature T cells were isolated from the lymph nodes and spleen, labeled with CellTrace Violet (Invitrogen), pretreated for 1 h with or without 50 μM zVAD-fmk, and stimulated with 5 μg/mL αCD3 and 1 μg/mL αCD28. At 48 h, cells were harvested, stained with PI, and analyzed by two-color flow cytometry (*n* = 3 independent experiments). **c** Western blot of cFLIP, Bcl-X_L_, and β-Actin in resting and stimulated mature T cells of indicated genotypes. Representative of three independent experiments. **d**, **e** Western blots of p65 phosphorylation, total p65, and β-Actin after stimulation of fibroblasts with **d** 10 ng/mL TNFα (*n* = 5 independent experiments) or **e** 1 μg/mL LPS (*n* = 3 independent experiments). Numbers under each gel indicate fold changes of signals for each lane

Given the pronounced defect in T cell development in the thymus and T-lymphopenia in the periphery in *Ripk1*^*−/−*^*Ripk3*^*−/−*^*Tradd*^*−/−*^ mice, we performed analysis of thymocytes for their ability to respond to stimuli through the DRs. WT, *Ripk1*^*−/−*^*Ripk3*^*−/−*^*Tradd*^*+/−*^, and *Ripk1*^*−/−*^*Ripk3*^*−/−*^*Tradd*^*−/−*^ thymocytes were similarly sensitive to Fas-induced cell death (Supplementary Fig. 7a). By contrast, *Ripk1*^*−/−*^*Ripk3*^*−/−*^*Tradd*^*+/−*^ thymocytes readily undergo TNFα-induced cell death (Fig. 4a). However, wild-type and *Ripk1*^*−/−*^*Ripk3*^*−/−*^*Tradd*^*−/−*^ thymocytes were equally resistant to TNFα. This is also true of mature peripheral T cells from the genotypes tested, which all undergo Fas-mediated apoptosis but only *Ripk1*^*−/−*^*Ripk3*^*−/−*^*Tradd*^*+/−*^ cells are sensitive to TNFα-induced cell death (Supplementary Fig. 7b, c). *Ripk1*^*−/−*^*Ripk3*^*−/−*^*Tradd*^*+/−*^ thymocytes are also sensitive to cell death induced by human TNFα, which binds to TNFR1 and not TNFR2^41^ (Supplementary Fig. 7d). In addition, blocking of TNFR1, but not TNFR2, largely prevents this cell death (Supplementary Fig. 7e). These data indicate that lack of RIPK1 sensitizes thymocytes to TRADD-mediated apoptosis induced by TNFα through TNFR1.

We also performed analysis of TCR-induced responses. The data show an increased PI^+^ population in *Ripk1*^*−/−*^*Ripk3*^*−/−*^*Tradd*^*−/−*^ TKO T cells after 48 h TCR stimulation, than wild type control T cells (Fig. 4b). Cell division barely occurred in *Ripk1*^*−/−*^*Ripk3*^*−/−*^*Tradd*^*−/−*^ TKO T cells stimulated through the TCR for 48 h, as compared to wild-type control T cells. A single wild-type allele of TRADD provided improvement, as indicated by an intermediate phenotype in *Ripk1*^*−/−*^*Ripk3*^*−/−*^*Tradd*^*+/−*^ T cells, which displayed moderately decreased cell death and moderately increased proliferation, when compared to *Ripk1*^*−/−*^*Ripk3*^*−/−*^*Tradd*^*−/−*^ TKO T cells (Fig. 4b). The defect in *Ripk1*^*−/−*^*Ripk3*^*−/−*^*Tradd*^*+/−*^ T cells remains significant when compared with wild-type T cells.

To determine the nature of the TCR-induced cell death, T cells were treated with the wide-spectrum caspase inhibitor zVAD-fmk. This treatment rescued *Ripk1*^*−/−*^*Ripk3*^*−/−*^*Tradd*^*−/−*^ T cells from death during TCR stimulation (Fig. 4b). Moreover, zVAD treatments greatly improved TCR-induced cell division/proliferation in *Ripk1*^*−/−*^*Ripk3*^*−/−*^*Tradd*^*−/−*^ T cells as well as in *Ripk1*^*−/−*^*Ripk3*^*−/−*^*Tradd*^*+/−*^ T cells (Fig. 4b). Therefore, these mutant TKO T cells undergo enhanced apoptosis in response to TCR stimulation, despite a resistance to TNFα-induced apoptosis (Fig. 4a). To understand further the enhanced apoptosis phenotype, western blotting analysis was performed to examine anti-apoptotic proteins. Freshly isolated resting *Ripk1*^*−/−*^*Ripk3*^*−/−*^*Tradd*^*−/−*^ T cells have lower expression of cFLIP than in *Ripk1*^*−/−*^*Ripk3*^*−/−*^*Tradd*^*+/−*^ and WT T cells (Fig. 4c). In contrast, Bcl-X_L_ is barely detectable in freshly isolated resting T cells, and is highly induced at 16 h post stimulation with anti-CD3/CD28 antibodies (Fig. 4c). This TCR-induced Bcl-X_L_ expression is severely impaired in *Ripk1*^*−/−*^*Ripk3*^*−/−*^*Tradd*^*−/−*^ T cells, as compared to *Ripk1*^*−/−*^*Ripk3*^*−/−*^*Tradd*^*+/−*^ and WT T cells (Fig. 4c). These two proteins are critical for T cell survival by inhibiting apoptosis and are target genes of the pro-survival NFκB pathway. *Ripk1*^*−/−*^*Ripk3*^*−/−*^*Tradd*^*−/−*^ T cells do appear to have reduced NFκB signaling, as indicated by reduced phosphorylation of NFκB p65 (Supplementary Fig. 8a). *Ripk1*^*−/−*^*Ripk3*^*−/−*^*Tradd*^*−/−*^ T cells also display elevated caspase activation upon stimulation (Supplementary Fig. 8b). Similarly, significantly elevated active caspase 3 levels were present in *Ripk1*^*−/−*^*Ripk3*^*−/−*^*Tradd*^*−/−*^ large intestine (Supplementary Fig. 8c). In total, a single wild-type *Tradd* allele is advantageous for *Ripk1*^*−/−*^*Ripk3*^*−/−*^ T cell survival after stimulation through the TCR. Moreover, the data clearly indicate that TRADD has a dual function in T cells.

### A critical role for TRADD in TNFα-mediated NFκB signaling

The ability to activate this pathway by TNFα was analyzed by examining induction of phosphorylation of NFκB p65 (p-p65) in mouse fibroblasts. In WT fibroblasts rapid phosphorylation of p65 was induced within 5 min of TNFα treatment, but NFκB activation is completely blocked in *Ripk1*^*−/−*^*Ripk3*^*−/−*^*Tradd*^*−/−*^ fibroblasts (Fig. 4d). A single allele of TRADD in *Ripk1*^*−/−*^*Ripk3*^*−/−*^*Tradd*^*+/−*^ fibroblasts results in an intermediate phenotype, that is a delayed activation of the NFκB pathway (Fig. 4d). We treated wild-type and mutant fibroblasts with LPS and analyzed NFκB induction. *Ripk1*^*−/−*^*Ripk3*^*−/−*^*Tradd*^*−/−*^ fibroblasts were capable of phosphorylating NFκB, albeit at slightly lower levels than WT fibroblasts (Fig. 4e). In total, TRADD is essential for TNFα-induced NFκB activation in *Ripk1*^*−/−*^*Ripk3*^*−/−*^ fibroblasts, but is dispensable for LPS-induced NFκB activation.

**Fig. 5:**
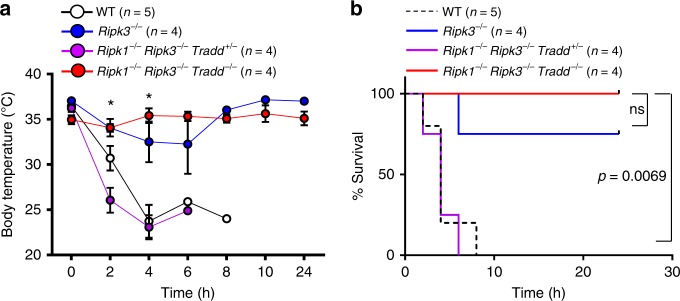
*Ripk1*^*−/−*^*Ripk3*^*−/−*^*Tradd*^*+/−*^ mice succumb to lethal TNFα-induced shock. **a** Body temperatures of mice of indicated genotypes over time. *t* tests show a significant difference in body temperature between *Ripk1*^*−/−*^*Ripk3*^*−/−*^*Tradd*^*+/−*^ and *Ripk1*^*−/−*^*Ripk3*^*−/−*^*Tradd*^*−/−*^ mice at 2 h and 4 h after injection. Graph presented as mean ± SEM. *p < 0.05 as determined by Student’s *t* test. **b** Kaplan–Meier plot showing survival of mice of indicated genotypes. Logrank test shows a significant difference in survival between *Ripk1*^*−/−*^*Ripk3*^*−/−*^*Tradd*^*+/−*^ and *Ripk1*^*−/−*^*Ripk3*^*−/−*^*Tradd*^*−/−*^ mice (*p* = 0.0069)

It is possible that the loss of DP thymocytes in *Ripk1*^*−/−*^*Ripk3*^*−/−*^*Tradd*^*−/−*^ (Fig. 3a) is secondary to the wasting disease (Fig. 1c). To this end, we analyzed younger mice (3 week old) which have no apparent wasting disease. The lymphoid organs of young *Ripk1*^*−/−*^*Ripk3*^*−/−*^*Tradd*^*−/−*^ mice are normal, as compared to WT controls (Supplementary Fig. 9a). There was no obvious defect in the thymus, and mature T cell numbers in lymph nodes was reduced in *Ripk1*^*−/−*^*Ripk3*^*−/−*^*Tradd*^*−/−*^ mice (Supplementary Fig. 9b, c). Thymocytes isolated from 3 week old *Ripk1*^*−/−*^*Ripk3*^*−/−*^*Tradd*^*−/−*^ mice are also resistant to TNFα-induced cell death unlike *Ripk1*^*−/−*^*Ripk3*^*−/−*^*Tradd*^*+/−*^ thymocytes (Supplementary Fig. 10), similar to the result in Fig. 4a. We performed analysis of NFκB target genes in TNFα-treated fibroblasts, and the data indicates impaired NFκB signaling in TKO cells. TNFAIP3 (or A20), an NFκB target gene, is downregulated in *Ripk1*^*−/−*^*Ripk3*^*−/−*^*Tradd*^*−/−*^ (TKO), compared to WT ear fibroblasts and is downregulated to a lesser degree in *Ripk1*^*−/−*^*Ripk3*^*−/−*^*Tradd*^*+/−*^ cells (Supplementary Fig. 11). We further analyzed in vivo response to TNFα treatment, by intravenous administration in mice. *Ripk1*^*−/−*^*Ripk3*^*−/−*^*Tradd*^*+/−*^ mice displayed a rapid drop in body temperature and succumbed to TNFα-induced shock, similar to WT mice (Fig. 5a, b). However, *Ripk1*^*−/−*^*Ripk3*^*−/−*^*Tradd*^*−/−*^ mice and *Ripk3*^*−/−*^ mice did not display a significant drop in body temperature and did not succumb to TNFα-induced shock (Fig. 5a, b).

## Discussion

The TRADD protein was identified almost a quarter century ago, as a potential adaptor molecule for TNFR1 signaling^35^. It was not until the first TRADD KO mice were generated in the year 2008 that the interrogation of the physiological function of TRADD became possible^37–39^. However, *Tradd*^*−/−*^ mice show no apparent developmental defect, which is in sharp contrast to the perinatal lethality in *Ripk1*^*−/−*^ mice^30^, and to the mid-gestation lethality in *Fadd*^*−/−*^ mice^23,24,26^. Furthermore, *Tradd*^*−/−*^ mice contain a normal lymphoid compartment, while conditional deletion of RIPK1 or FADD results in T lymphopenia in the periphery^31,42^. These observations appear to indicate TRADD being dispensable during mouse development and lymphocyte development.

Despite having no impact on mouse development, deletion of the necroptosis effector protein kinase RIPK3 completely rescues *Fadd*^*−/−*^ mice from embryonic lethality, and the resulting *Fadd*^*−/−*^*Ripk3*^*−/−*^ DKO mice show progressive lymphoproliferative disease after reaching adulthood^29^. The fact that absence of TRADD is unable to rescue the embryonic defect in *Fadd*^*−/−*^ mice (Supplementary Fig. 2) indicates that TRADD is dispensable for necroptotic signaling in vivo. Previous studies have indicated a potential role for TRADD in necroptosis, as indicated by *Tradd*^*−/−*^ MEFs being resistant to treatment with TNFα, cycloheximide and zVAD (TCZ)^38^. This observation also contrasts the normal embryogenesis in *Fadd*^*−/−*^*Ripk1*^*−/−*^ mice^26^. Therefore, necroptosis in embryonic cells is TRADD-independent and occurs directly via RIPK1 in vivo when FADD-mediated apoptosis is inactivated.

Mice lacking the adaptor kinase RIPK1 die shortly after birth, and blocking necroptosis through deletion of the downstream RIPK3, or blocking apoptosis via deletion of FADD provides little survival advantage, because the resulting *Fadd*^*−/−*^*Ripk1*^*−/−*^ or *Ripk1*^*−/−*^*Ripk3*^*−/−*^ DKO mice die within several days after birth^26,29,32,33^. Only when both RIPK3 and FADD are deleted do *Ripk1*^*−/−*^ mice survive to adulthood^29,32,33^. The striking observation in the current study that deletion of TRADD rescues *Ripk1*^*−/−*^*Ripk3*^*−/−*^ mice from perinatal lethality (Fig. 1) indicates that TRADD, similar to FADD, may mediate apoptosis in neonatal *Ripk1*^*−/−*^*Ripk3*^*−/−*^ mice and cause perinatal death. Therefore, a lack of phenotype in *Tradd*^*−/−*^ mice is due in part to the presence of RIPK1.

Although both FADD and TRADD mediate apoptosis, functional distinction between these two adaptors is evident. Unlike *Ripk1*^*−/−*^*Ripk3*^*−/−*^*Fadd*^*−/−*^ mice, which have normal postnatal development, *Ripk1*^*−/−*^*Ripk3*^*−/−*^*Tradd*^*−/−*^ mice show greatly diminished post-weaning survival (Fig. 1b). The lethality in young adulthood may be explained in part by the severe defect in the intestine in *Ripk1*^*−/−*^*Ripk3*^*−/−*^*Tradd*^*−/−*^ mice (Fig. 1e). The elevated Caspase 3 activation is indicative of enhanced apoptosis in the intestine when both alleles of *Tradd* are inactivated in *Ripk1*^*−/−*^*Ripk3*^*−/−*^ mice (Fig. 1e). Interestingly, these symptoms are reversed by retaining a single wild-type allele of *Tradd* in *Ripk1*^*−/−*^*Ripk3*^*−/−*^ mice (Fig. 2), contrasting heterozygosity of FADD which is not sufficient to prevent lethality in *Ripk1*^*−/−*^*Ripk3*^*−/−*^ mice during perinatal development^29^. While reducing apoptosis in the intestine, the rescue through haplosufficiency of TRADD did not lead to lymphadenopathy and splenomegaly in the resulting *Ripk1*^*−/−*^*Ripk3*^*−/−*^*Tradd*^*+/−*^ mice (Supplementary Fig. 4), unlike the severe *lpr*-like symptom in *Ripk3*^*−/−*^*Fadd*^*−/−*^ or *Ripk1*^*−/−*^*Ripk3*^*−/−*^*Fadd*^*−/−*^ mice (Supplementary Fig. 4)^29,33^.

In addition to the intestinal defect, *Ripk1*^*−/−*^*Ripk3*^*−/−*^*Tradd*^*−/−*^ TKO mice display severe T lymphopenia (Fig. 3). Interestingly, like in wild-type mice, TKO thymocytes and peripheral mature T cells are resistant to cell death triggered by TNFα (Fig. 4a and Supplementary Fig. 7,b). However, presence of one allele of wild-type TRADD rendered *Ripk1*^*−/−*^*Ripk3*^*−/−*^ thymocytes and mature T cells hypersensitive to TNFα-induced death (Fig. 4a and Supplementary Fig. 7b, d). In total, the data argue that TRADD indeed mediates TNFα-induced apoptosis in T cells, when RIPK1 is absent. Therefore, T lymphopenia in the *Ripk1*^*−/−*^*Ripk3*^*−/−*^*Tradd*^*−/−*^ mice is counterintuitive, given that thymocytes from these TKO mice are completely resistant to TNFα-induced apoptosis. This paradox can be explained in part by the defect in TCR-induced survival and proliferative responses in peripheral mature *Ripk1*^*−/−*^*Ripk3*^*−/−*^*Tradd*^*−/−*^ TKO T cells (Fig. 4b). More importantly, TRADD haplosufficiency, as observed in *Ripk1*^*−/−*^*Ripk3*^*−/−*^*Tradd*^*+/−*^ mice, indicates a dual role for TRADD in signaling TCR-induced survival and proliferative responses (Fig. 4b).

The severely impaired proliferative responses to TCR stimulation in *Ripk1*^*−/−*^*Ripk3*^*−/−*^*Tradd*^*−/−*^ TKO mice appear to be due largely to caspase-dependent apoptosis, because the wide-spectrum caspase inhibitor zVAD suppressed TCR-induced death in *Ripk1*^*−/−*^*Ripk3*^*−/−*^*Tradd*^*−/−*^ TKO T cells and improved cell division/proliferation (Fig. 4b and Supplementary Fig. 8,b). It is important to iterate that death receptor-induced apoptosis is not affected by deletion of TRADD, as the TKO T cells behave similarly to wild-type T cells in response to stimulation of Fas and TNFR1 (Fig. 4a and Supplementary Fig. 7a–c). Therefore, the result in Fig. 4b implies a role for TRADD in TCR-induced survival signaling, beyond its role in promoting TNFR1-induced apoptosis as it does in perinatal cells.

One possible explanation for the improved survival of *Ripk1*^*−/−*^*Ripk3*^*−/−*^*Tradd*^*+/−*^ cells and mice is the requirement for TRADD in NFκB signaling. Indeed, we found that dermal fibroblasts of adult TKO mice show a complete blockage of TNFα-mediated NFκB signaling as shown by lack of phosphorylation of p65 (Fig. 4d). This indicates that TRADD is essential for NFκB signaling in the absence of RIPK1. Due to the severe defects in the intestines and T cells of TKO mice, there may be a cell type-specific requirement for TRADD-mediated NFκB signaling to prevent apoptosis. Further supporting this notion is the observation that TKO T cells have lower expression of the NFκB targets, cFLIP and Bcl-X_L_, as compared with *Ripk1*^*−/−*^*Ripk3*^*−/−*^*Tradd*^*+/−*^ or wild-type cells (Fig. 4c). In total, similar to RIPK1 and FADD, TRADD can also initiate or protect against cell death depending on the conditions and cell type.

In summary, the findings in the current study identify a previously unappreciated role for TRADD during perinatal development and in adult mice lacking RIPK1 and RIPK3. Gene dosage of TRADD elicits a Goldilocks effect on the survival of *Ripk1*^*−/−*^*Ripk3*^*−/−*^ mice, whereby *Tradd*^*+/+*^ and *Tradd*^*−/−*^ results in death by apoptosis while haplosufficiency of TRADD proves to be optimal for survival. These findings demonstrate the interplay between cell death and NFκB signaling, identify TRADD as a key component in cell death regulation, and provide insight into the workings of the apoptotic pathway in the presence and absence of RIPK1.

## Methods

### Mice

Both males and females of *mus musculus* in a C57BL/6 background were used. *Tradd*^*−/−*^ mice were provided by Liu^38^. *Ripk1*^*+/−*^ mice were provided by Kelliher^30^. *Ripk3*^*−/−*^ mice were provided by Newton and Dixit^43^. All mice were bred and housed in a specific pathogen free environment at Thomas Jefferson University. After intercrosses of indicated mice, neonates were monitored daily for survival from birth to weaning age and deceased pups were immediately collected and genotyped. Viable pups were genotyped at weaning age and monitored for survival. Kaplan–Meier survival curves were generated using Prism software (Graphpad Software, Inc.). All animal studies are approved by the Institutional Animal Care and Use Committee (IACUC) at Thomas Jefferson University. For genotyping, DNA was isolated from the ear of mice with an alkaline lysis solution containing 25 mM NaOH and 0.2 mM EDTA followed by neutralization with 40 mM Tris-HCl pH 5.0. Allele specific primers were used to amplify wild type and knockout alleles as previously described for *Fadd*^23^ (WT primers: 5′- ATG GAC CCA TTC CTG GTG CTG CTG-3′; 5′-CAG TAG ATC GTG TCG GCG CAG CG-3′ and KO primers: 5′-ACTGTAGTGCCCAGCAGAGACCAGC -3′; 5′-CGCTCGGTGTTCGAGGCCACACGC-3′); *Ripk1*^30^ (WT&KO primers: 5′-CTGCTAAAGCGCATGCTC-3′; 5′-TGTGTCAAGTCTCCCTGCAG-3′; 5′-CACGGTCCTTTTGCCCTG-3′); *Tradd*^38^ (WT primers: 5′-CACTGACTCTTCAAGACCAGCAGAC-3′; 5′-CAACAGATCCTCTGCTAGACTAGTG-3′ and KO primers: 5′-TGCACATGTGTCCTCGAGTG-3′; 5′-GGAGAGCTTGGCTGTCTTGG-3′); *Ripk3*^43^ (WT&KO primers: 5′-CGCTTTAGAAGCCTTCAGGTTGAC-3′; 5′-GCCTGCCCATCAGCAACTC-3′; 5′-CCAGAGGCCACTTGTGTAGCG-3′).

### Embryo analysis

Mating of *Fadd*^*+/−*^*Tradd*^*−/−*^ mice was setup in the evening and females were checked in the morning. When vaginal plug was detected, embryos were designated E0.5. Embryos were isolated from pregnant mice at E12.5-E18.5 and genotyped as described above.

### Cell culture including fibroblast and T cell preparation

Thymocytes and peripheral T cells purified from spleen and lymph nodes were cultured in complete RPMI media (cRPMI) containing 10% FBS, 2 mM L-Glutamine, penicillin (100 U/mL), streptomycin (100 μg/mL), and β-mercaptoethanol (50 μM). Fibroblasts were isolated from the ears of euthanized adult mice was adapted from previously established protocol^44^. In summary, ears were sterilized with 70% ethanol and cut into small pieces. Ear pieces were incubated with warm 0.25% trypsin at 37 °C, 5% CO_2_ for 90 min, then broken up and passed through a cell strainer with complete DMEM (cDMEM) containing 10% FBS, 1mM L-Glutamine, penicillin (100 U/mL), streptomycin (100 μg/mL), and β-mercaptoethanol (50 μM). Isolated cells were washed once in cDMEM and cultured on tissue culture-treated plates at 37 °C, 5% CO_2_.

### Histology

Intestines were prepared for histology using the swiss roll technique followed by fixation in 10% formalin for 24 h. Samples were washed in PBS, placed in 70% ethanol, and then embedded in formalin. Sections were stained with hematoxylin and eosin or with anti-cleaved caspase 3 antibody (Cell Signaling Technology #9661).

### Flow cytometry

Spleen, lymph nodes, and thymus were isolated during mouse dissection. Bone marrow was isolated from tibias and femurs of mice. Single cell suspension was prepared and red blood cells were depleted by hypotonic lysis. Cells were stained on ice for 30 minutes in PBS containing 3% BSA, 1 mM EDTA, and 0.05% sodium azide with fluorochrome-conjugated antibodies anti-CD3 (BD Biosciences, clone 17A2), anti-CD4 (BD Biosciences, clone GK1.5), anti-CD8 (Caltag, clone CT-CD8a), anti-B220 (Caltag, clone RA3-6B2), anti-IgM (Jackson ImmunoResearch, 115-116-075), anti-Mac1 (BD Biosciences, clone M1/70), anti-Gr1 (eBioscience, clone RB6-8C5). Samples were washed twice with PBS. Data was acquired on LSR II flow cytometric analyzer (BD Biosciences) and analyzed using FlowJo software (Treestar). Total cell numbers were counted on Countess Automated Cell Counter (Invitrogen) and lymphocyte cellularity was determined by multiplying the total cell number of indicated organ by percentage of CD3^+^ or B220^+^ cells within the organ obtained by flow cytometry.

### Cell death assays

Thymocytes (10^5^) or purified mature peripheral T cells were seeded in triplicate in 96 well flat-bottom plates in cRPMI with various concentrations of anti-Fas antibody (BD Biosciences, clone Jo2), mouse TNFα (Alexis Pharmaceuticals), or human TNFα (Life Technologies) for 16 h with cycloheximide. In indicated experiments, thymocytes were pre-treated with TNFR1 or TNFR2 blocking antibodies (Life Technologies) in complete RPMI on ice for 4 h before TNFα treatment. Mature peripheral T cells were treated with FLAG-tagged sFasL (Alexis Pharmaceuticals) and anti-FLAG M2 antibody (1 μg/mL, Sigma). After incubation, 1 μg/mL propidium iodide (PI) and the percentage of cell death was analyzed by flow cytometry as determined by PI^+^ cells.

### T cell proliferation assays

Mature T cells were purified of the spleen and lymph nodes by staining with anti-Thy1.2 antibody (BD Biosciences, clone 53–2.1) and sorting Thy1.2^+^ cells using a FACS Aria (BD Biosciences). For fluorescent labeling, purified T cells (10^6^/mL) were labeled with 1 μM CellTrace^TM^ Violet in PBS for 20 min at 37 °C, 5% CO_2_. cRPMI (5 mL) was added to each tube and incubated for an additional 5 min at 37 °C 5% CO_2_. Labeled cells were then centrifuged for 5 minutes, supernatant was removed, and cells were resuspended in cRPMI. Labeled T cells (2 × 10^5^) were seeded in 96 well round bottom plates pre-coated with 5 μg/mL anti-CD3 (BD Biosciences, clone 145-2C11) in cRPMI with 1 μg/mL anti-CD28 antibodies (BD Biosciences, clone 37.51). At indicated time points, T cells were collected, 1 μg/mL PI was added, and cells were analyzed by two-color flow cytometry on the LSR II (BD Biosciences) for cell death and division kinetics. Data was analyzed using FlowJo software (Treestar). Caspase activities in purified mature T cells was measured using CellEvent^TM^ Caspase 3/7 Green Flow Cytometry Assay Kit (Life Technology), per the manufacturer’s instructions. Briefly, T cells were stimulated with 5 μg/mL anti-CD3 and 1 μg/mL anti-CD28 antibodies for the given time points. CellEvent Caspase 3/7 Green Detection Reagent was added to T cell culture, followed by 30 min incubation at 37 °C, 5% CO_2_. Samples were immediately run on LSR II cytometer (BD Biosciences) and data analyzed using FlowJo Software (Treestar).

### Western blotting analysis

To prepare samples for western blot, single cell suspension of splenocytes was prepared from mice of indicated genotypes to detect the presence or absence of RIPK1, RIPK3, and TRADD. T cells were enriched from the spleen and lymph nodes of mice of indicated genotypes by negative selection using MojoSort Mouse CD3 T cell Isolation kit (Biolegend, #480031) and stimulated with 5 μg/mL anti-CD3 and 1 μg/mL anti-CD28 antibodies in cRPMI for 16 h or with 1x eBioscience Cell Stimulation Cocktail of PMA/Ionomycin (Invitrogen) for 5 min. Fibroblasts were stimulated with 10 ng/mL TNFα or 1 μg/mL LPS for indicated time points. Cell lysates were prepared in cold RIPA lysis buffer (50 mM Tris pH 8.0, 150 mM NaCl, 1% Nonidet P-40, 0.5% deoxycholate, 0.1% SDS, 1 mM phenylmethyl sulphonyl fluoride (PMSF), and 1X Halt protease inhibitor cocktail (Thermo Scientific). Additional inhibitors were added when probing for phospho-specific proteins (50 mM β-glycerophosphate, 1 mM sodium vanadate, 10 mM sodium fluoride). Proteins (40 μg) were separated on a 10% SDS-PAGE gel and transferred to a nitrocellulose membrane. Membranes were blocked with 5% milk in TBST and blotted with antibodies specific for RIPK1 (BD Biosciences, #610459), RIPK3 (ProSci, #2283), TRADD (Santa Cruz, sc7868), cFLIP (Alexis Biochemicals, ALX-804-127), Bcl-X_L_ (Cell Signaling #2764), total p65 (Cell Signaling #3034), or β-Actin (Sigma Aldrich, A-5441) in 5% milk in TBST at 4 °C overnight. For phospho-specific antibodies, membranes were blocked with 5% BSA in TBST for 1 h at room temperature and blotted overnight with anti-phosphorylated p65 (Cell Signaling Technology, #3033) in 5% BSA in TBST. Membranes were stained with HRP-conjugated secondary antibodies at room temperature for 1 h. Membranes were treated with Western Lightning Plus-ECL (Perkin Elmer, NEL105001EA) and developed using X-Ray film or FluorChem M Western Blot Imaging machine (ProteinSimple). Quantification of signals on X-ray films was performed using the ImageJ software. Uncropped gels are provided in the Source Data file.

### TNFα-induced shock

Ten micrograms of TNFα (Sino Biological) was injected intravenously into mice. Mice were monitored for survival and core body temperature every 2 h for 10 h and at 24 h. Core body temperature was taken with a MicroTherma 2T Hand Held thermometer with rectal probe (Braintree Scientific). Mice were euthanized if moribund or body temperature dropped below 23.6 °C.

### Statistical analysis

Data is represented as mean ± standard error of the mean (SEM). Student’s *t*-tests were performed using Prism software (Graphpad Software, Inc.) to determine *p* value. A *p* value < 0.05 was considered significant. Logrank tests were performed to compare survival of various genotypes in Kaplan Meier plots. Chi square analysis was performed to determine significance of genotype tables.

### Reporting summary

Further information on experimental design is available in the Nature Research Reporting Summary linked to this article.

## Supplementary information

Source Data

Supplementary Information

Reporting Summary

## Data Availability

The data that support the findings of this study are available within the Article and its Supplementary Information or, on reasonable request, from the corresponding author.
