# Supplementary material for: TRADD regulates perinatal development and adulthood survival in mice lacking RIPK1 and RIPK3
Source: Nat Commun. 2019 Feb 11;10:705. doi: 10.1038/s41467-019-08584-5 (PMC6370879; doi:10.1038/s41467-019-08584-5)
Supplement: Supplementary file 2 — Supplementary Information [file 41467_2019_8584_MOESM2_ESM.pdf]

Supplementary Information

TRADD regulates perinatal development and adulthood survival in mice lacking RIPK1 and RIPK3

Dowling et al.

Supplementary Figure 1

a

Offspring of *Fadd*$^{+/-}$ *Tnf*$\alpha^{-/-}$ intercrosses

Genotype	*Fadd*<br>*Tnf*α	+/+<br>-/-	+/-<br>-/-	-/-<br>-/-	Total
E12.5-16.5 Embryos	Actual	13	23	**15**	51
	Expected	13	26	13	52
E17.5-18.5 Embryos	Actual	20	40	**25**	85
	Expected	20	40	20	80
After Birth	Actual	21	23	**0**	44
	Expected	21	42	21	84

b

E18.5

Tnf$\alpha^{-/-}$ *Fadd*$^{-/-}$ *Tnf*$\alpha^{-/-}$

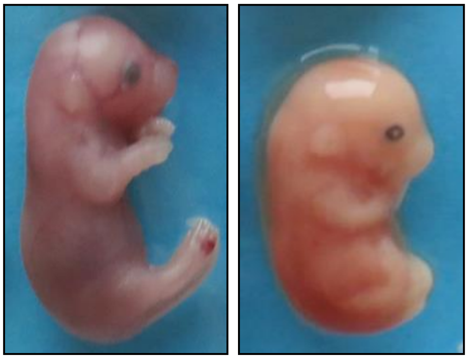

c

Offspring of *Fadd*$^{+/-}$ *Tnf*$\alpha^{+/-}$ intercrosses

Genotype	*Fadd*<br>*Tnf*α	+/+, +/-<br>+/+, +/-, -/-	-/-<br>+/+, +/-	-/-<br>-/-	Total
E17.5-18.5 Embryos	Actual	25	**0**	**3**	28
	Expected	25	6	2	33

Supplementary Figure 1. Deletion of TNFα improves *Fadd*$^{-/-}$ embryogenesis **a**, Actual and expected frequencies of embryos of various embryonic stages from intercrosses of *Fadd*$^{+/-}$ *Tnf*$\alpha^{-/-}$ mice. Chi square analysis shows no significant difference between actual and expected frequencies at E12.5-E16.5 (p = 0.721) or E17.5-E18.5 (p = 0.535), but there is a significant difference after birth (p < 0.0001). **b**, Embryos with indicated genotypes at E18.5, (n = 3 independent experiments). **c**, Actual and expected frequencies of embryos from *Fadd*$^{+/-}$*Tnf*$\alpha^{+/-}$ intercrosses. Chi square analysis shows a significant difference at late embryonic stages (p = 0.0388).

Supplementary Figure 2

a Offspring of *Fadd*$^{+/-}$ *Tradd*$^{-/-}$ intercrosses

Genotype	*Fadd*<br>*Tradd*	+/+<br>-/-	+/-<br>-/-	-/-<br>-/-	Total
E12.5 Embryos	Actual	8	21	**2**	31
	Expected	8	16	8	32
E13.5-18.5 Embryos	Actual	27	55	**0**	82
	Expected	27	54	27	108

b

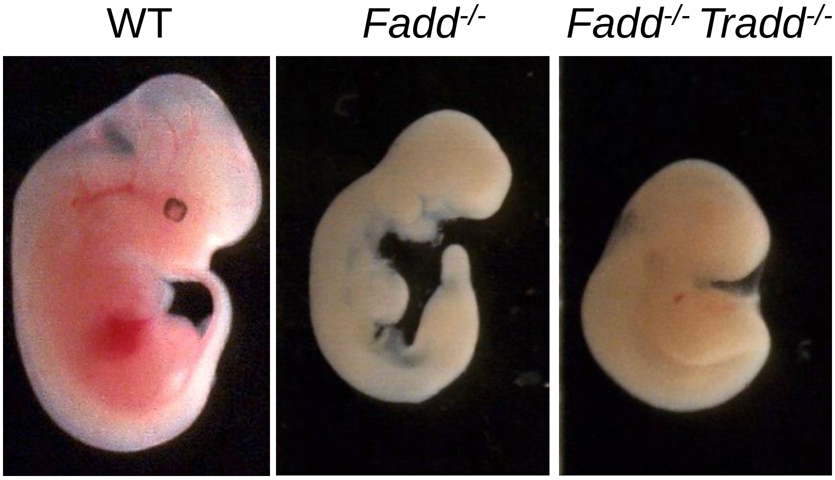

Supplementary Figure 2. TRADD does not mediate necroptosis in *Fadd*$^{-/-}$ embryos a, Actual and expected frequencies of embryos of various embryonic stages from intercrosses of *Fadd*$^{+/-}$*Tradd*$^{-/-}$ mice. Chi square analysis shows a significant difference between actual and expected frequencies at E12.5 ($p = 0.048$) and E13.5-18.5 ($p < 0.0001$). **b,** Embryos with indicated genotypes at E12.5 (n = 3 independent experiments).

Supplementary Figure 3

a Offspring of *Ripk1*$^{+/-}$ *Fadd*$^{+/-}$ *Tradd*$^{-/-}$ intercrosses

Genotype	*Ripk1* / *Fadd* / *Tradd*	+/+, +/- / +/+, +/- / -/-	+/+, +/- / -/- / -/-	-/- / +/+, +/- / -/-	-/- / -/- / -/-	Total
At birth	Actual	93	0	4	3	100
	Expected	93	31	31	10	165
At weaning age (3 wk)	Actual	89	0	0	0	89
	Expected	90	30	30	10	160

b

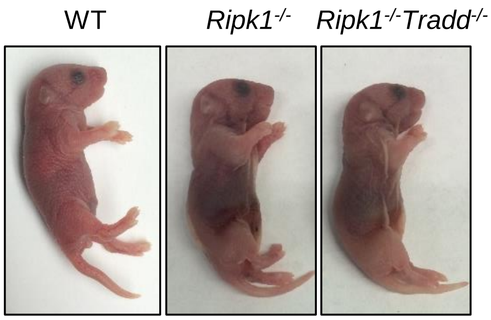

c

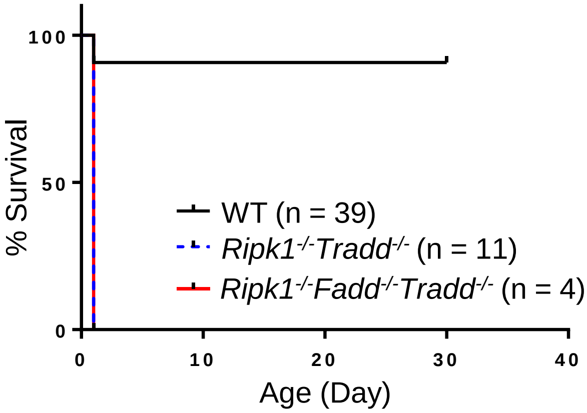

Supplementary Figure 3. Deletion of TRADD does not rescue perinatal lethality of *Ripk1*$^{-/-}$ or *Ripk1*$^{-/-}$*Fadd*$^{-/-}$ mice **a**, Actual and expected frequency of indicated genotypes at birth and weaning age from intercross of *Ripk1*$^{+/-}$*Fadd*$^{+/-}$*Tradd*$^{-/-}$ mice. Chi square analysis shows a significant difference between actual and expected frequencies ($p < 0.0001$). **b**, Newborn mice of indicated genotypes (n = 4 independent experiments). **c**, Kaplan-Meier plot showing survival of mice of indicated genotypes. Logrank test shows no significant difference in survival between *Ripk1*$^{-/-}$*Fadd*$^{-/-}$*Tradd*$^{-/-}$ and *Ripk1*$^{-/-}$*Tradd*$^{-/-}$mice ($p > 0.999$).

Supplementary Figure 4

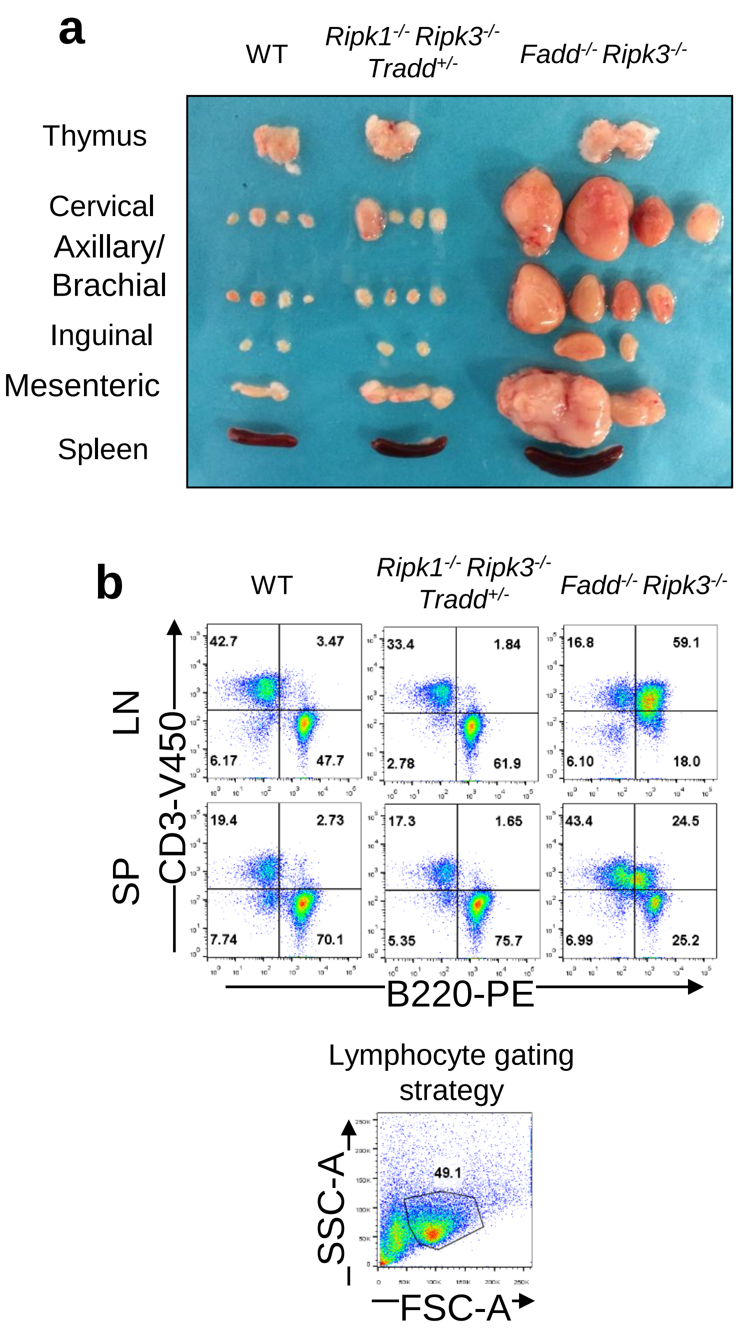

Supplementary Figure 4. Analysis of aged *Ripk1*$^{-/-}$*Ripk3*$^{-/-}$*Tradd*$^{+/-}$ mice a, Spleen, lymph nodes, and thymus of 9 month old mice of indicated genotypes (n = 4 independent experiments). **b**, Two-color flow cytometric plots investigating the presence of T cells ($CD3^{+}B220^{-}$) and B cells ($CD3^{-}B220^{+}$) and abnormal T cells ($CD3^{+}B220^{+}$) in the lymph nodes and spleen of aged mice (n = 4 independent experiments)

Supplementary Figure 5

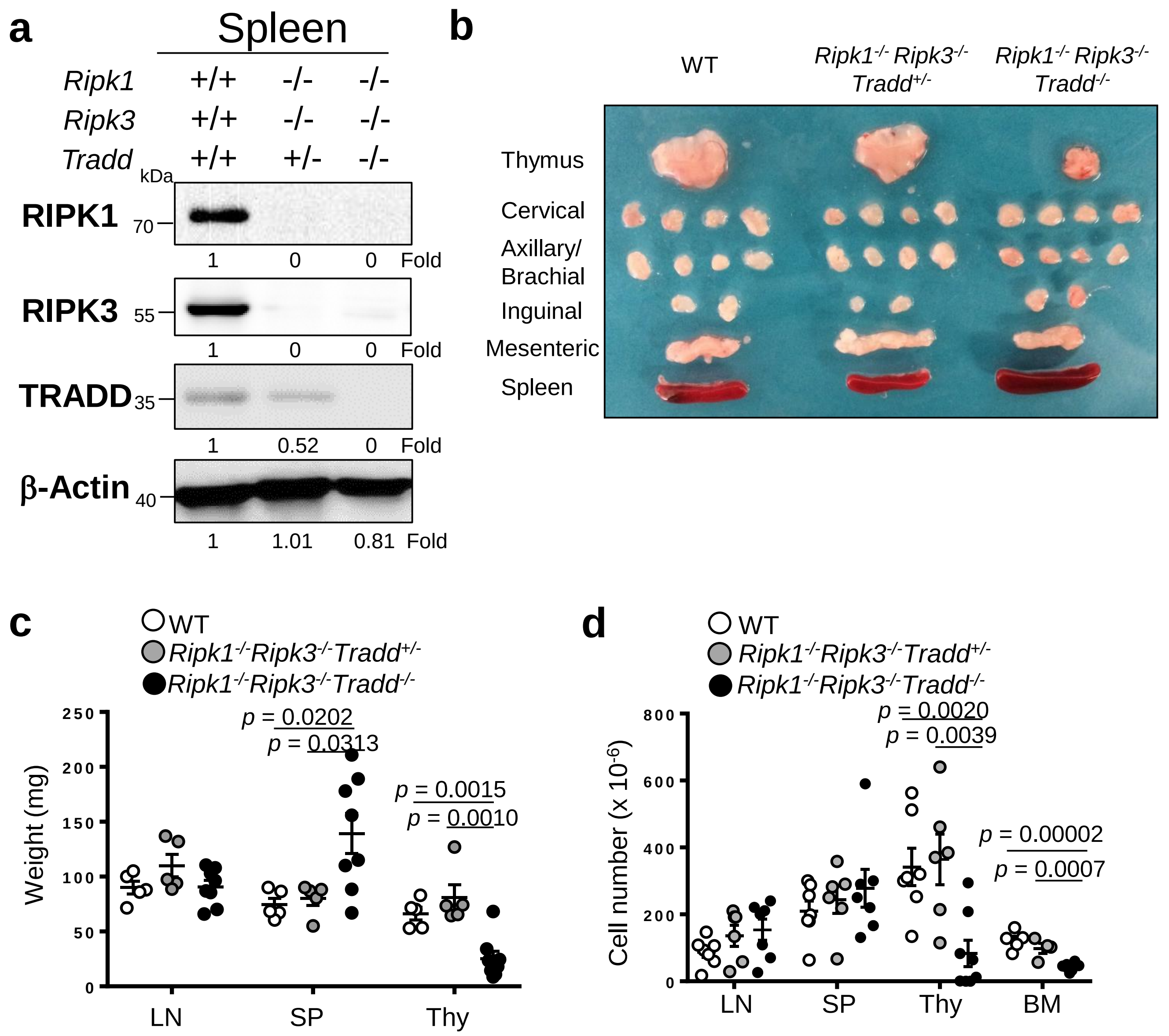

Supplementary Figure 5. Lymphoid compartment of *Ripk1$^{-/-}$Ripk3$^{-/-}$Tradd$^{-/-}$* mice a, Western blots showing RIPK1, RIPK3, TRADD, and β-Actin protein concentrations in the spleen (n = 3 independent experiments). **b**, Spleen, lymph nodes, and thymus of 7 week old mice of indicated genotypes (n = 5 independent experiments). **c**, Weight of lymph nodes, spleen, and thymus of 7-8 week old mice (n = 6 independent experiments). **d**, Total cellularity of lymphoid organs of 7-8 week old mice (n = 6 independent experiments). Errors in **c** and **d** represent mean±SEM.

Supplementary Figure 6

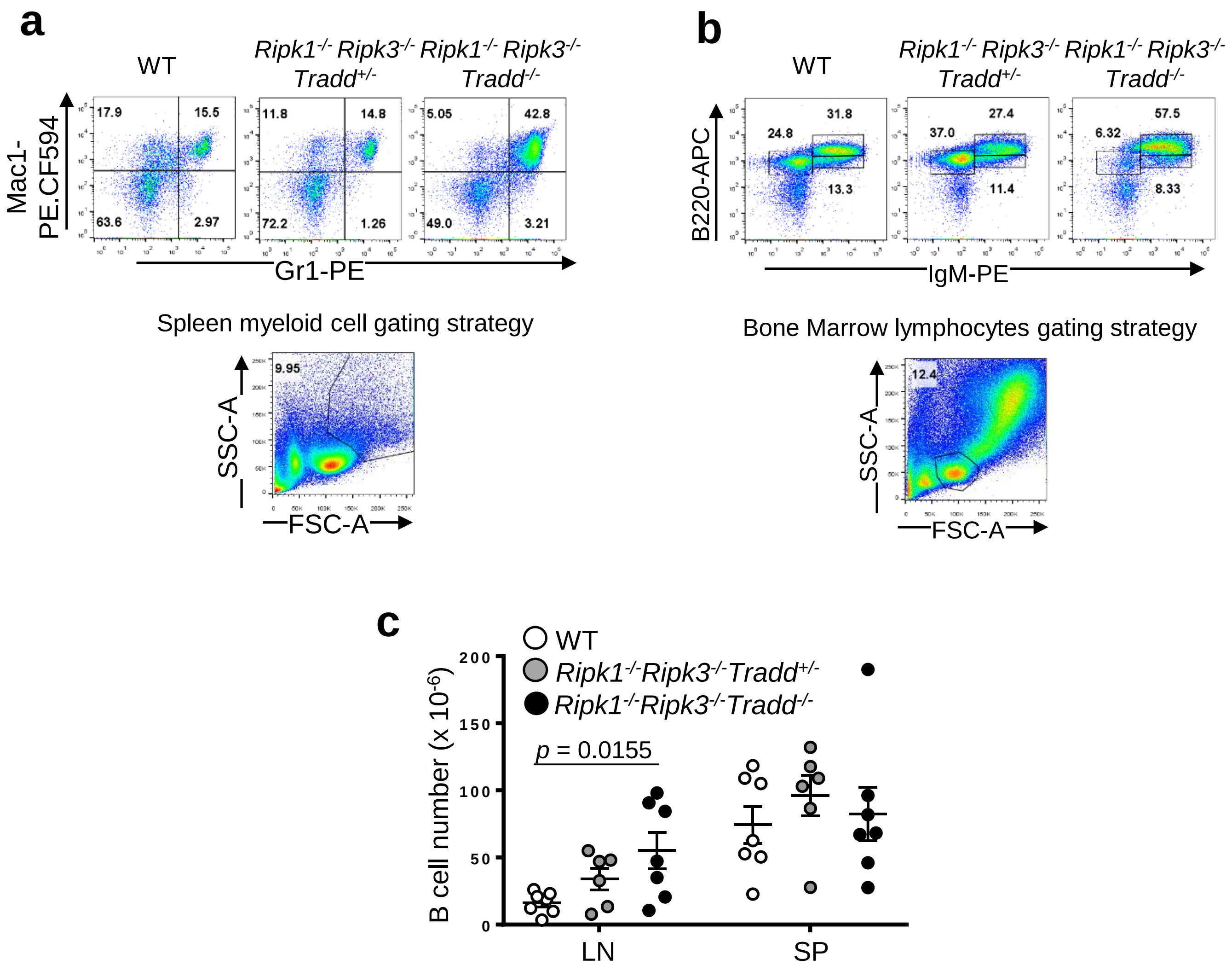

Supplementary Figure 6. Myeloid cells and B cell in *Ripk1$^{-/-}$Ripk3$^{-/-}$Tradd$^{-/-}$* mice a, Myeloid populations in the spleen identified by Mac-1 and Gr-1 staining (n = 4 independent experiments). **b**, Representative 2-color flow cytometric plots showing B220^{+}IgM^{-} pre/pro B cells, B220^{+}IgM^{+} immature B cells, and B220^{hi}IgM^{+} mature B cells in the bone marrow (n = 4 independent experiments). **c**, B220^{+} B cell numbers in the lymph nodes and spleen (n = 7 independent experiments). All graphs presented as mean ± SEM.

Supplementary Figure 7

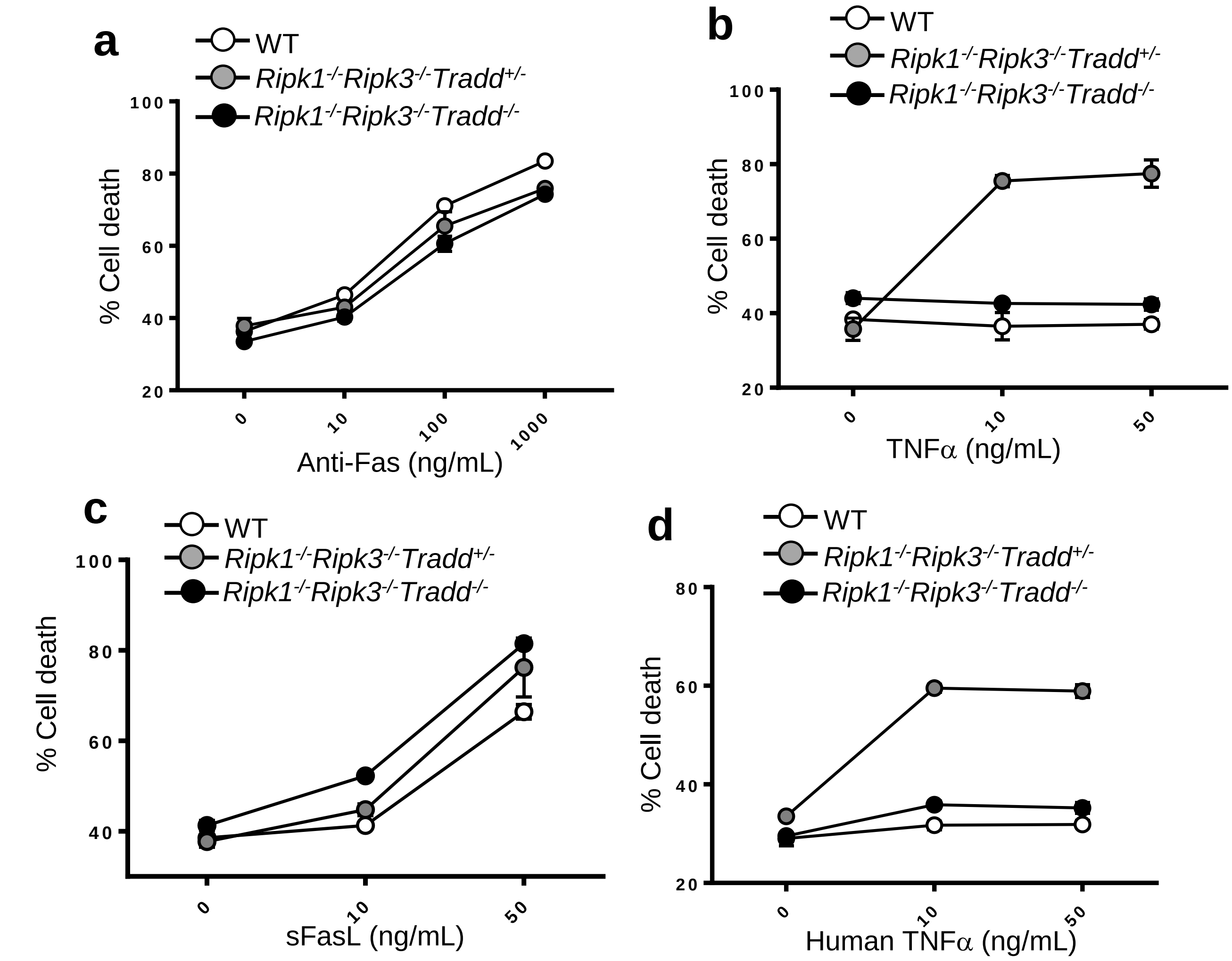

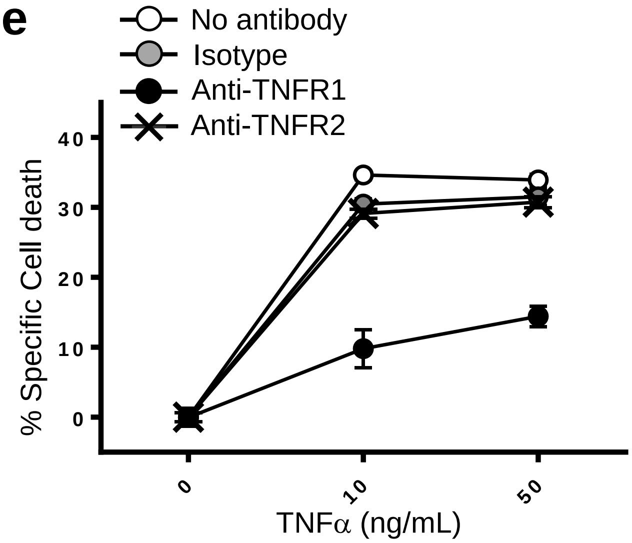

Supplementary Figure 7. Cell death in T cells a, Thymocytes were treated with indicated concentrations of anti-Fas antibody for 16 h with 30 μg/mL cycloheximide. **b,** Peripheral mature T cells were treated with indicated concentrations of TNFα for 16 h with 1 μg/mL cycloheximide. **c**, Peripheral mature T cells treated with indicated concentrations of sFasL and 1 μg/mL anti-FLAG. **d**, Thymocytes were treated with indicated concentrations of human TNFα for 16 h with 30 μg/mL cycloheximide. **e,** Thymocytes from *Ripk1$^{-/-}$ Ripk3$^{-/-}$Tradd$^{+/-}$* mice were pre-treated with indicated blocking antibodies (10 μg/mL) and then treated with indicated concentrations of TNFα. After addition of 1 μg/mL propidium iodide (PI), dead cells (PI^{+}) were determined by flow cytometry. Errors, ±SEM of triplicates. Data represent three independent experiments.

Supplementary Figure 8

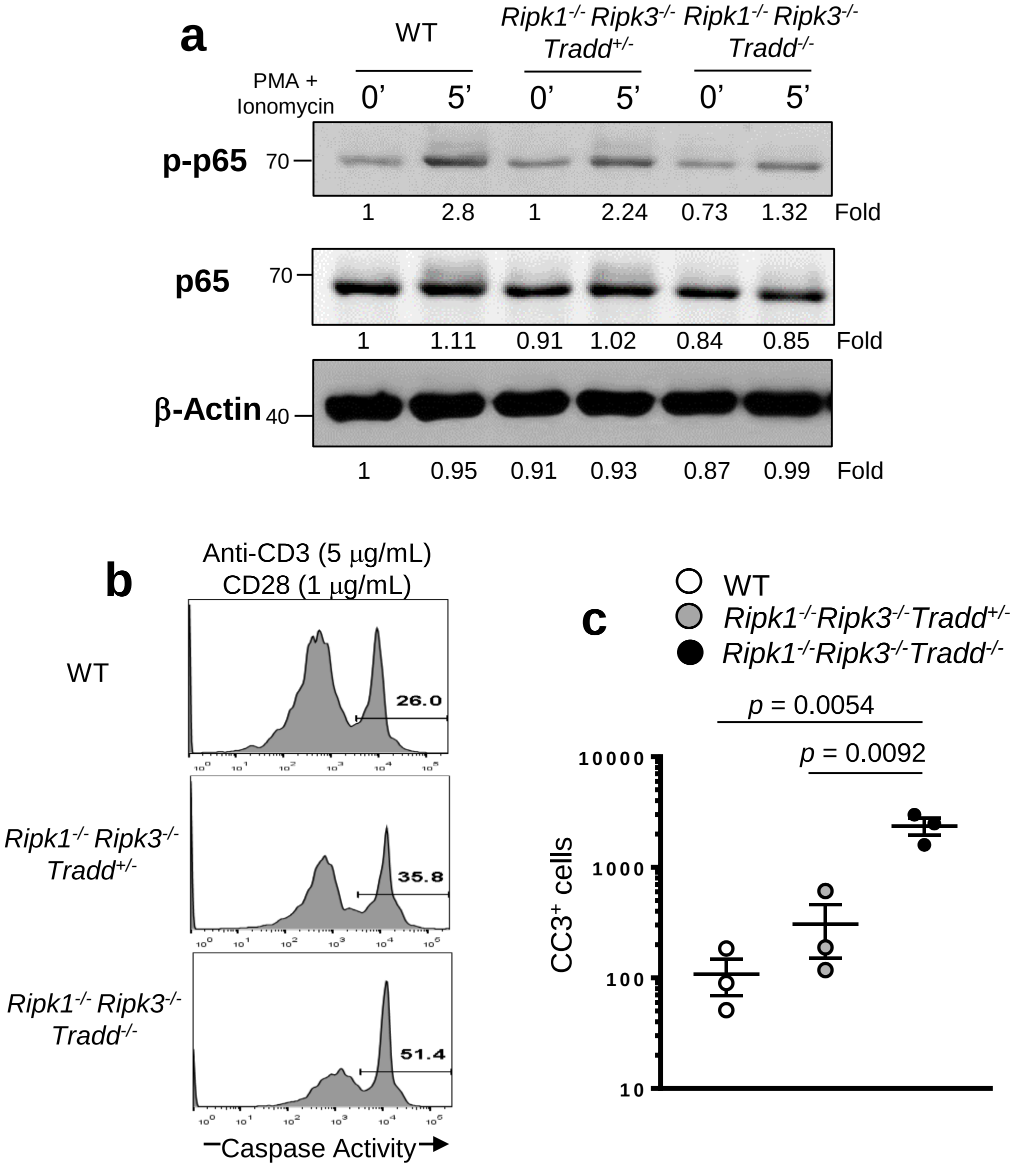

Supplementary Figure 8. NFκB signaling and caspase activity analysis a, Western blot analysis of NFκB p65 phosphorylation, total p65, after stimulation of peripheral mature T cells. The data represents three independent experiments. β-Actin, loading control. **b**, Caspase activities of mature T cells after 48 h stimulation was measured by flow cytometry with CellEvent Caspase 3/7 Green Flow Reagent. **c**, Scattered dot plot showing quantification of CC3$^+$ cells in the large intestine sections using the ImageScope software (Leica Biosystems). Errors, mean±SEM, n = 3.

Supplementary Figure 9

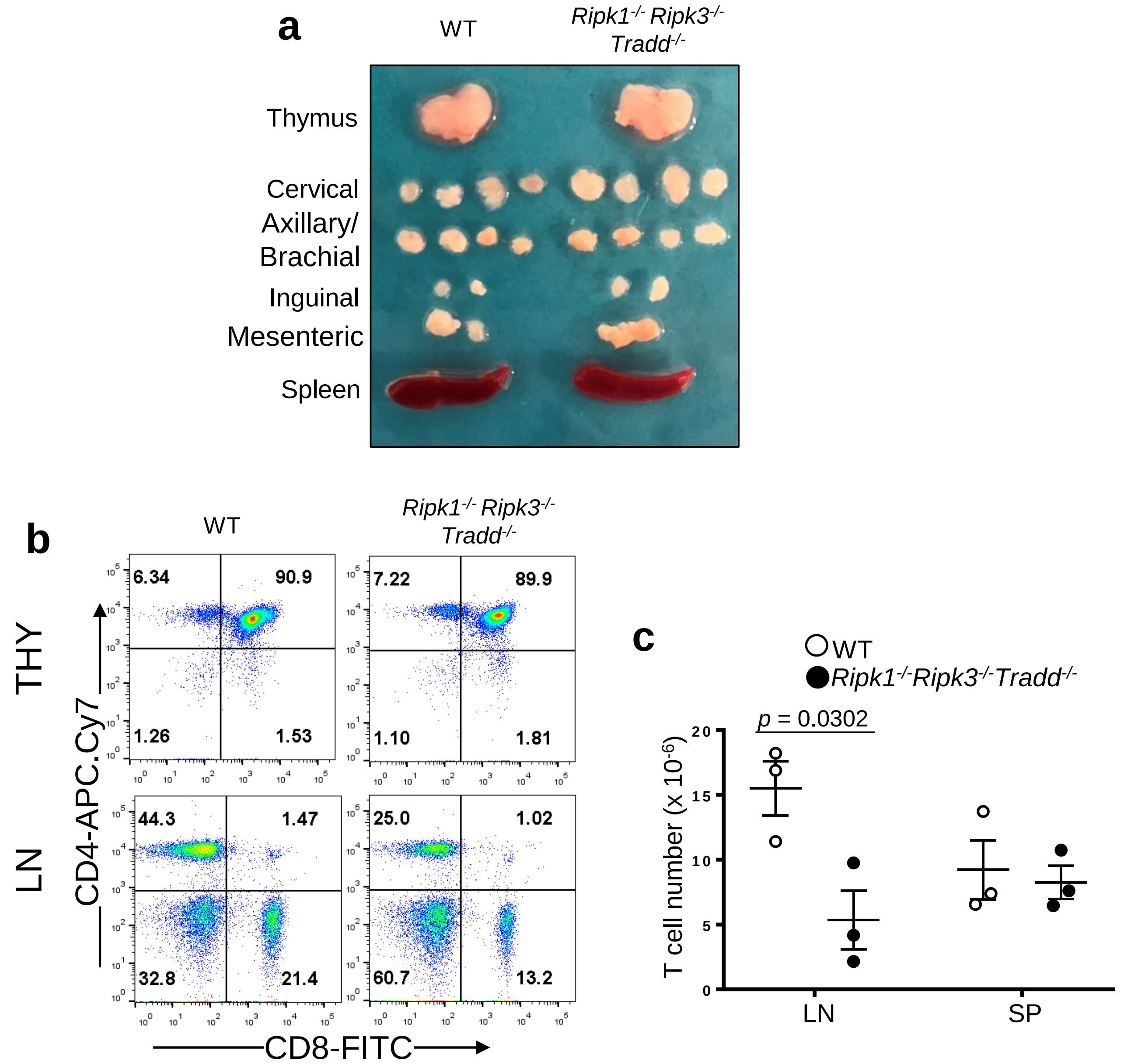

Supplementary Figure 9. Analysis of weaning age *Ripk1$^{-/-}$Ripk3$^{-/-}$Tradd$^{-/-}$* mice a, The spleen, lymph nodes, and thymus of 3 week old mice of indicated genotypes. Data are representative of at least three mice of each genotype. **b** Two-color flow cytometric plots showing CD4^{+} and CD8^{+} populations in the thymus and lymph nodes of three week old mice (representative of three independent experiments). **c**, CD3^{+} T cell numbers in the lymphoid organs of three week old mice of indicated genotypes (n = 3 independent experiments)**.** Errors, mean±SEM.

Supplementary Figure 10

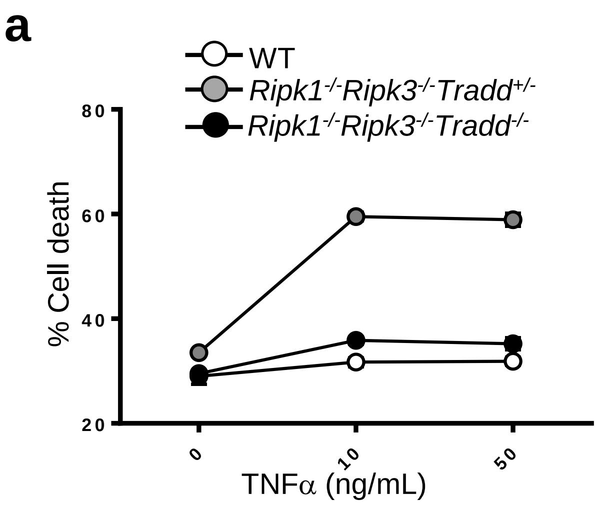

Supplementary Figure 10. Cell death in thymocytes from young mice a, Thymocytes isolated from 3 week old mice were treated with indicated concentrations of TNFα for 16 h with 30 μg/mL cycloheximide. Cell death analysis was performed by PI staining using a flow cytometer. Data represents three independent experiments. Errors, ± SEM.

Supplementary Figure 11

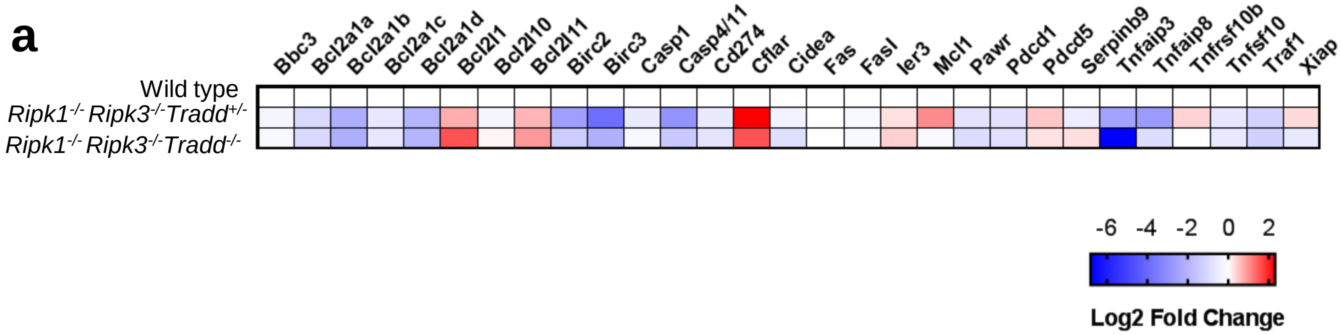

b

	WT	*Ripk1$^{-/-}$Ripk3$^{-/-}$ Tradd$^{+/-}$*	*Ripk1$^{-/-}$Ripk3$^{-/-}$ Tradd$^{-/-}$*
**Bbc3**	5.08	4.79	4.89
**Bcl2a1a**	5.69	4.66	4.66
**Bcl2a1b**	6.66	4.47	4.47
**Bcl2a1c**	4.01	3.35	3.35
**Bcl2a1d**	6.44	4.42	4.46
**Bcl2l1**	11.36	12.1	12.91
**Bcl2l10**	3.8	3.51	3.89
**Bcl2l11**	4.8	5.47	5.72
**Birc2**	9.8	7.21	8.41
**Birc3**	11.34	7.43	9.21
**Casp1**	4.99	4.42	4.78
**Casp4/11**	7.86	4.96	6.41
**Cd274**	5.12	4.55	4.39
**Cflar**	8.66	10.97	10.21
**Cidea**	4.65	4.32	3.78
**Fas**	6.73	6.73	6.73
**Fasl**	3.3	3.1	3.1
**Ier3**	11.5	11.77	11.92
**Mcl1**	11.87	12.91	11.7
**Pawr**	8.67	7.9	7.73
**Pdcd1**	4.36	3.54	3.54
**Pdcd5**	11.71	12.21	11.97
**Serpinb9**	11.25	10.76	11.54
**Tnfaip3**	13.27	10.77	6.45
**Tnfaip8**	8.14	5.45	7.21
**Tnfrsf10b**	11.77	12.16	11.77
**Tnfsf10**	3.79	3.1	3.16
**Traf1**	6.38	5.15	5.14
**Xiap**	11.39	11.72	10.79

Supplementary Figure 11. NFκB target gene expression a, Heatmap of RNA expression analysis from dermal fibroblasts stimulated with 10 ng/mL TNFα for 4 h. Data expressed as fold change relative to wild type mouse fibroblasts. **b,** Gene expression levels of the array analysis are shown Log2 signal intensity.
