# Supplementary material for: TRADD regulates perinatal development and adulthood survival in mice lacking RIPK1 and RIPK3
Source: Nat Commun. 2019 Feb 11;10:705. doi: 10.1038/s41467-019-08584-5 (PMC6370879; doi:10.1038/s41467-019-08584-5)
Supplement: Supplementary file 3 — Reporting Summary [file 41467_2019_8584_MOESM3_ESM.pdf]

nature research

Corresponding author(s): Jianke Zhang

Reporting Summary

Nature Research wishes to improve the reproducibility of the work that we publish. This form provides structure for consistency and transparency in reporting. For further information on Nature Research policies, see Authors & Referees and the Editorial Policy Checklist.

Statistical parameters

When statistical analyses are reported, confirm that the following items are present in the relevant location (e.g. figure legend, table legend, main text, or Methods section).

n/a	Confirmed	
☐	☒	The exact sample size (n) for each experimental group/condition, given as a discrete number and unit of measurement
☐	☒	An indication of whether measurements were taken from distinct samples or whether the same sample was measured repeatedly
☐	☒	The statistical test(s) used AND whether they are one- or two-sided<br>*Only common tests should be described solely by name; describe more complex techniques in the Methods section.*
☒	☐	A description of all covariates tested
☒	☐	A description of any assumptions or corrections, such as tests of normality and adjustment for multiple comparisons
☐	☒	A full description of the statistics including central tendency (e.g. means) or other basic estimates (e.g. regression coefficient) AND variation (e.g. standard deviation) or associated estimates of uncertainty (e.g. confidence intervals)
☐	☒	For null hypothesis testing, the test statistic (e.g. F, t, r) with confidence intervals, effect sizes, degrees of freedom and P value noted<br>*Give P values as exact values whenever suitable.*
☒	☐	For Bayesian analysis, information on the choice of priors and Markov chain Monte Carlo settings
☒	☐	For hierarchical and complex designs, identification of the appropriate level for tests and full reporting of outcomes
☒	☐	Estimates of effect sizes (e.g. Cohen's d, Pearson's r), indicating how they were calculated
☐	☒	Clearly defined error bars<br>*State explicitly what error bars represent (e.g. SD, SE, CI)*

Our web collection on statistics for biologists may be useful.

Software and code

Policy information about availability of computer code

Data collection	Flow Cytometry data was collected using FACSDiva Software 8.0.1 (BD)
Data analysis	Flow cytometry data was analyzed using FlowJo software 10.0.7 (Treestar). Graphs were made using Graphpad Prism 7.03.

For manuscripts utilizing custom algorithms or software that are central to the research but not yet described in published literature, software must be made available to editors/reviewers upon request. We strongly encourage code deposition in a community repository (e.g. GitHub). See the Nature Research guidelines for submitting code & software for further information.

Data

Policy information about availability of data

All manuscripts must include a data availability statement. This statement should provide the following information, where applicable:

- Accession codes, unique identifiers, or web links for publicly available datasets
- A list of figures that have associated raw data
- A description of any restrictions on data availability

The authors declare that all data supporting the findings of this study are available within the paper and its supplementary information.

Field-specific reporting

Please select the best fit for your research. If you are not sure, read the appropriate sections before making your selection.

☒ Life sciences ☐ Behavioural & social sciences ☐ Ecological, evolutionary & environmental sciences

For a reference copy of the document with all sections, see nature.com/authors/policies/ReportingSummary-flat.pdf

Life sciences study design

All studies must disclose on these points even when the disclosure is negative.

Sample size	The sample sizes for each experiment are included in the associated figure legend. No calculation was used to determine sample size prior to experiments.
Data exclusions	No data were excluded
Replication	For each set of experiments, results were repeated at least 3 times. Exact number of replicates indicated for each experiment in corresponding figure legend.
Randomization	Mice were placed into groups based on genotype
Blinding	Researchers were not blinded during collection or analysis as the Ripk1-/-Ripk3-/-Tradd-/- mice were clearly distinguishable from other controls

Reporting for specific materials, systems and methods

Materials & experimental systems

n/a	Involved in the study	
☒	☐	Unique biological materials
☐	☒	Antibodies
☒	☐	Eukaryotic cell lines
☒	☐	Palaeontology
☐	☒	Animals and other organisms
☒	☐	Human research participants

Methods

n/a	Involved in the study	
☒	☐	ChIP-seq
☐	☒	Flow cytometry
☒	☐	MRI-based neuroimaging

Antibodies

Antibodies used

Flow Cytometry:
CD3-V450: BD Biosciences, Cat#561389, Lot# 5337554, Clone 17A2, Dilution 1/300
B220-PE: Caltag, Cat# RM2604, Lot# 13011203, Clone RA3-6B2, Dilution 1/1000
CD4-APC.Cy7: BD Biosciences, Cat# 552051, Lot# 5135831, Clone: GK1.5, Dilution 1/500
CD8-FITC: Caltag, Cat# RM2201, Lot# 22010904, Clone: CT-CD8a, Dilution 1/1000
Thy1.2-FITC: BD Biosciences, Cat# 553004, Lot# 3218552, Clone 53-2.1, Dilution 1/1000
Mac1-PE.CF594: BD Biosciences, Cat# 562287, Lot# 2181893, Clone: M1/70, Dilution 1/1000
Gr1-PE: eBiosciences, Cat# 12-5931-82, Lot# E021655, Clone: RB6-8C5, Dilution 1/500
B220-APC: BD Biosciences, Cat# 553092, Lot# 5139848, Clone RA3-6B2, Dilution 1/500
IgM-PE: Jackson Immunoresearch Laboratories, Cat# 115-116-075, Lot# 78473, polyclonal, Dilution 1/500

Cell Death Experiments:
Anti-CD120a (TNFR1): Life Technologies, Cat# 16-1202-81, Lot# 2029686
Anti-CD120b (TNFR2): Life Technologies, Cat# 14-1203-81, Lot# 2003176
Anti-FLAG M2: Sigma Aldrich, Cat# F-3165, Lot# 128H9200

Histology:
Cleaved Caspase 3: Cell Signaling Technology, Cat# 9661L, Lot# 43, polyclonal, Dilution 1/500

Western blotting:
RIP1: BD Biosciences, Cat# 610459, Lot# 38587, Clone: 38/RIP, Dilution 1/1000
RIP3: ProSci, Cat# 2283, Lot# 2283-0704, polyclonal, Dilution 1/1000

TRADD: Santa Cruz, Cat# sc7868, Lot#C3010, polyclonal, Dilution 1/1000
cFLIP: Alexis Biochemicals, 804-127-C100, Lot# L20682, Clone: DAVE-2, Dilution, 1/4000
Bcl-xL: Cell Signaling Technology, Cat# 2764, Lot# 1, Clone: 54H6, Dilution 1/1000
p-p65: Cell Signaling Technology, Cat# 3033L, Lot# 6, Clone: 93H1, Dilution 1/1000
p65: Cell Signaling Technology, Cat# 3034, Lot #5, polyclonal, Dilution 1/1000
beta-Actin: Sigma Aldrich, Cat# A5441, Clone: AC-15, Dilution 1/2000
HRP-conjugated Goat anti-mouse: Vector Laboratories, Cat# PI-2000, Lot# X0328, Dilution 1/10000
HRP-conjugated Goat anti-rabbit: Vector Laboratories, Cat# PI-1000, Lot# X0126, Dilution 1/10000
HRP-conjugated Goat anti-Rat: GE Healthcare, Cat# NA935V, Lot# 354130, Dilution 1/10000

Validation	All antibodies have been validated by the respective companies and information is available within the Data Sheets for those antibodies.

Animals and other organisms

Policy information about studies involving animals; ARRIVE guidelines recommended for reporting animal research

Laboratory animals	All animals used were Mus musculus males and females. The majority of experiments used either 3 week old or 7-8 week old mice at time of analysis with exception of Extended Data Fig. 4 which used 9 month old mice for lpr disease characterization and embryos used in Extended Data Fig. 1,2.
Wild animals	n/a
Field-collected samples	n/a

Flow Cytometry

Plots

Confirm that:

☒ The axis labels state the marker and fluorochrome used (e.g. CD4-FITC).

☒ The axis scales are clearly visible. Include numbers along axes only for bottom left plot of group (a 'group' is an analysis of identical markers).

☒ All plots are contour plots with outliers or pseudocolor plots.

☒ A numerical value for number of cells or percentage (with statistics) is provided.

Methodology

Sample preparation	Single cell suspensions were stained in PBS with 3% BSA, 1mM EDTA, and 0.05% sodium azide for given antibody dilutions for 30 minutes. Then samples were washed twice with PBS.
Instrument	LSR II (BD Biosciences) and BD FACSAria (BD Biosciences)
Software	FACSDiva Software 8.0.1 (BD), FlowJo 10.0.7 (Treestar)
Cell population abundance	Purity of sorted T cells was determined by measuring Thy1.2-FITC+ cells and were >95% pure.
Gating strategy	For characterization of the lymphoid organs, cells were gated by FSC/SSC based on size and granularity for lymphocytes (for T and B cell analysis) or myeloid cells (Mac1/Gr1 staining). For Celltrace experiments of purified T cells, the population shown is ungated.

☒ Tick this box to confirm that a figure exemplifying the gating strategy is provided in the Supplementary Information.
